# Hyperthermic intraperitoneal chemotherapy combined with systemic chemotherapy for gastric cancer peritoneal carcinomatosis: A protocol for systematic review and meta-analysis of randomized controlled trials

**DOI:** 10.1097/MD.0000000000020973

**Published:** 2020-07-02

**Authors:** Yidan Lu, Zheng Jin, Song Zheng, Yurong Bai, Yangcheng Sun

**Affiliations:** a Affiliated Hangzhou First People's Hospital, Zhejiang Chinese Medical University; b Hangzhou Cancer Hospital; c Affiliated Hangzhou First People's Hospital, Zhejiang University School of Medicine; d Affiliated Hangzhou First People's Hospital, Nanjing Medical University, Hangzhou, China.

**Keywords:** gastric cancer peritoneal carcinomatosis, hyperthermic intraperitoneal chemotherapy, meta-analysis, protocol, systemic chemotherapy

## Abstract

**Background:**

The prognosis of gastric cancer peritoneal carcinomatosis (GCPC) remains poor despite recent advances in systemic chemotherapy (SC) with an average survival less than 6 months. Current evidence supporting the utility of hyperthermic intraperitoneal chemotherapy (HIPEC) combined with SC for GCPC is limited. We plan to provide a systematic review and meta-analysis of randomized controlled trials to evaluate the comparative effects and safety of HIPEC combined with SC in the management of GCPC.

**Methods:**

Randomized controlled trials evaluating HIPEC combined with SC versus SC as first-line treatment for GCPC will be searched in MEDLINE, EMBASE, Web of Science, the Cochrane Library, ClinicalTrials.gov, and Google Scholar, from database inception to April 30, 2020. Data on study design, participant characteristics, intervention details, and outcomes will be extracted. Primary outcomes to be assessed are: median progression-free survival; secondary outcomes are: median survival time, 1- year survival rate, 2-year survival rate, objective response rate, and adverse events. Meta-analysis will be performed using RevMan V.5.3 statistical software. Data will be combined with a random effect model. Study quality will be assessed using the Cochrane Risk of Bias Tool. Heterogeneity will be assessed, and if necessary, a subgroup analysis will be performed to explore the source of heterogeneity.

**Results:**

The results will provide useful information about the effectiveness and safety of HIPEC combined with systemic chemotherapy regimens in patients with gastric cancer peritoneal carcinomatosis.

**Conclusion:**

The findings of the study will be disseminated through peer-reviewed journal.

**The registration number:**

INPLASY202050006.

**DOI number:**

10.37766/inplasy2020.5.0006.

**Key Points**

- This is the first meta-analysis comparing hyperthermic intraperitoneal chemotherapy combined with systemic chemotherapy versus systemic chemotherapy for gastric cancer peritoneal carcinomatosis.
- A conceivable subgroup analysis of specific patient characteristics make it possible to identify the area that may require further research.
- The study selection, data extraction, and quality assessment will be conducted independently by 2 investigators.
- A possible weakness may be the quality of the trials we include.

## 1 Introduction

Gastric cancer is among the most frequently occurring malignancies worldwide, as the fourth/fifth most common malignancy and the second leading cause of cancer-related deaths, with only about 25% of patients surviving for 5 years.^[1,2]^ Most patients with gastric cancer are in advanced stages when diagnosed, and peritoneal carcinomatosis is the most frequent site of metastasis.^[3,4]^ Systemic chemotherapy (SC) could improve the survival of patients with gastric metastatic cancer for 7 to 10 months.^[5]^ However, for patients with gastric cancer peritoneal carcinomatosis (GCPC), SC had limited efficacy. Peritoneal barrier makes the effective intraperitoneal concentration hard to achieve and only a small fraction of the systemically administered drug is delivered to the peritoneum.^[6]^ The prognosis of GCPC remains poor despite recent advances in SC with an average survival less than 6 months.^[7–10]^

In the1980s, Koga et al^[11–13]^ and Fujimoto et al^[14]^ applied hyperthermic intraperitoneal chemotherapy (HIPEC) to the treatment of gastric cancer. HIPEC is a technique characterized by the infusion of high concentration chemotherapy drugs at high temperature. Some studies had demonstrated for patients with advanced gastric cancer without peritoneal metastasis, radical resection combined with HIPEC can prevent patients from peritoneal metastasis and prolong the survival.^[15–18]^ For peritoneal metastasis, as a local manifestation of gastric cancer, a systemic disease, the comprehensive treatment mode based on SC is still adopted. A meta-analysis demonstrated that HIPEC combined with cytoreduction surgery had a beneficial effect on response in patients with advanced gastric cancer and GCPC.^[19]^ Zhao et al demonstrated that HIPEC combined with basic care of SC could extend the survival period, improve the quality of life, benefit the patients with advanced gastric cancer.^[20]^ The GYMSSA Trial (NCT00941655)^[21]^ indicated that HIPEC compared with SC, maximal cytoreduction surgery can achieve prolonged survival in selected patients with GCPC. But the sample size was only 16.

However, the current evidence supporting the efficacy of HIPEC combined with SC as first-line treatment for GCPC is limited. Therefore, we will conduct a meta-analysis to make comprehensive evaluations and compare HIPEC + SC with SC in patients with GCPC.

## 2 Methods

According to the recommendations specified in the Cochrane Handbook for Intervention Reviews^[22]^ we will perform the review and report it following the Preferred Reporting Items for Systematic Reviews and Meta-Analyses statement.^[23]^

### 2.1 Criteria for considering studies for this review

Eligibility criteria are formed in terms of the patients, interventions, comparison and outcomes (PICO) structure described below. Randomized controlled trials (RCTs) will be included in this study according to the criteria:

### 2.2 Patients (P)

Patients who had been pathologically diagnosed as gastric adenocarcinoma/gastroesophageal junction adenocarcinoma with peritoneal metastasis will be included.

### 2.3 Interventions/comparison (I-C)

The comparison of interventions will be designed as HIPEC + SC versus SC. In the HIPEC + SC groups, there should be a specific description of HIPEC with description of the drugs, regimens and the thermotherapy techniques used, including but not limited to: “intraperitoneal paclitaxel combined hyperthermia,” “intraperitoneal hyperthermic perfusion,” “hyperthermic peritoneal perfusion,” and so on; All the studies on single intraperitoneal chemotherapy or hyperthermia including “early postoperative intraperitoneal chemotherapy” and “normal intraperitoneal chemotherapy” will be excluded. In SC groups, the procedures described as “ SC,” “oral chemotherapy,” or “intravenous chemotherapy” for GCPC will be included.

### 2.4 Type of outcomes (O)

The primary outcome will be median progression-free survival. The secondary outcomes will be median survival time, 1-year survival rate, 2-year survival rate objective response rate, and adverse events.

### 2.5 Study design

Only RCTs will be included. Exclusion criteria will be:

(1) non-RCTs, single-arm, unreasonable control setting;
(2) observational studies, case reports, reviews, editorials and letters to editor;
(3) duplicate studies, in vitro studies or animal studies, and
(4) no full-text, no data on any of the primary or secondary outcomes.

Unpublished trials and abstracts will be included if the methodology and data are accessible.

### 2.6 Search methods for identification of studies

Two investigators (YL and YB) will independently search the following electronic health databases: Medline (by PubMed, from database inception to April 30, 2020), Embase (by Elsevier), and Controlled Clinical Trials of the Cochrane Collaboration (Cochrane Central Register of Controlled Trials). We have used the following MeSH search terms and their synonyms: “hyperthermic intraperitoneal chemotherapy,” “stomach,” “gastric cancer,” “carcinosis,” “peritoneal carcinomatosis,” “comparative study,” “randomized controlled trials,” “prospective study,” and “comparative study.” To further increase the robustness of the literature search, we will recursively search references of relevant primary or secondary studies to identify additional eligible studies. If any up-to-date evidence is published during the review period, we will evaluate the eligibility of each study and consider its addition to the analysis. Endnote software will be utilized to remove duplicates. We will use predetermined inclusion criteria to estimate the eligibility of retrieved articles by title and abstract. We will review and consider the full-text if this information is insufficient for qualification assessment.

### 2.7 Data collection and analysis

#### 2.7.1 Selection of studies

**Figure 1:**
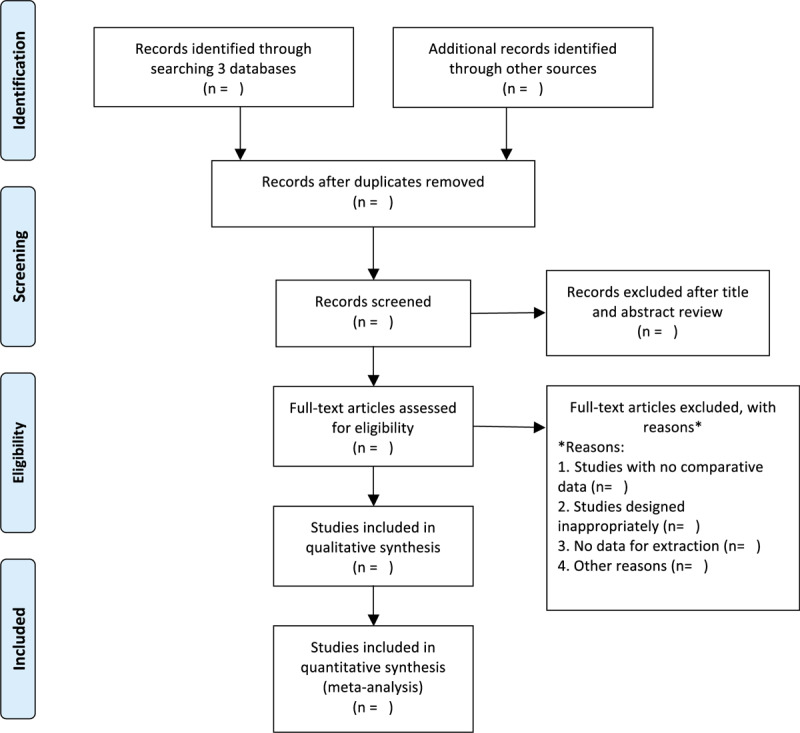
Flow diagram of the study selection process.

The 2 investigators will evaluate the title and summary independently (YL and ZJ) and will select studies that conform to the criteria to review the full-text, and then subsequently assess the adequacy to the proposed PICO criteria. In case of disagreement, a consensus meeting will take place before the final decision. The study selection procedure is illustrated in a Preferred Reporting Items for Systematic Reviews and Meta-Analyses flow chart (Fig. 1).

#### 2.7.2 Data extraction and management

All the records retrieved from the databases are processed through EndNote X9. All extracted data are. Two investigators (YB and YS) will extract relevant data independently from all studies and store in a Microsoft Excel spreadsheet. The data included study features, population characteristics (title, author, publication time, research type, control setting) data required for quality assessment, and the different outcome measures. Population characteristics include age, gender, tumor pathologic variables, chemotherapy regimen, and specific details of intervention measures. We will communicate with the authors for missing information. If it does not work, we will analyze the available data, when possible, from other available statistics, such as *P*-values and could only exclude the studies without data on any of the primary or secondary outcomes.

#### 2.7.3 Assessment of risk of bias in included studies

Methodological quality will be assessed by 2 individual investigators (YL and ZJ) based on the Cochrane Collaboration's tool for assessing the risk of bias.^[24]^ The tool appraises existence of selection bias which considers the following 5 domains for each outcome evaluated:

(1) bias arising from the randomization process,
(2) bias due to deviations from intended interventions,
(3) bias due to missing outcome data,
(4) bias in the measurement of the outcome, and
(5) bias in the selection of the reported result, and attrition and reporting bias by evaluating incomplete and selective data reporting.

Each project is assigned a judgement of high, low, or unclear risk. In case of disagreement, a discussion between the reviewers will take place before the final classification.

### 2.8 Data synthesis

Review Manager (RevMan) (Version 5.3. Copenhagen: The Nordic Cochrane Centre, The Cochrane Collaboration, 2014) was used to perform all the statistical analyses. Hazard ratios will be calculated for survival data. If there is no direct data, we will make an effort to extract data from Kaplan–Meier curves based on Parmaretal method.^[25]^ Risk ratios will be calculated for discrete variables. Owning to the assumption of inherently various study scenarios (HIPEC patterns, systematic chemotherapy regimens), a random-effects model will be assumed. When sufficient studies (≥10) are included in the analysis of primary outcomes, we will construct funnel plots to evaluate publication bias. Otherwise, Egger test will be applied.^[26]^ Heterogeneity among studies will be assessed by calculating the *I*^2^ statistics^[27]^ whereby *I*^*2*^ < 25% indicates no heterogeneity, 25% ≤ *I*^*2*^ < 50% indicates mild heterogeneity, 50% ≤ *I*^*2*^ < 75% indicates moderate heterogeneity and *I*^*2*^ ≥ 75% indicates large heterogeneity. If substantial heterogeneity (<50%) is observed, subgroup analysis using the following variables may be performed to explore the source of heterogeneity: chemotherapy regimens; gender; grade of cancer; anatomical position; combination mode of intraperitoneal chemotherapy and hyperthermia.

### 2.9 Patient and public involvement

Given that the data collected in this systematic review and meta-analysis originate from previously published studies, patients and the general public were not involved in the process of research issues or outcome metrics that we want to evaluate.

## 3 Discussion

Most patients with peritoneal carcinomatosis of digestive tract origin die within 6 months.^[28]^ National Comprehensive Cancer Network guidelines recommend peritoneal cytology be managed with SC or with supportive care.^[29]^ The poor success of SC is likely because of the peritoneal-plasma barrier.^[30]^ Intraperitoneal chemotherapy enables intraperitoneal tumors to be exposed to high concentrations of drugs, without increasing the blood concentration to toxic levels.^[31]^ And hyperthermia accelerates blood circulation, thereby improving local drug concentration in the tumor so that the heat and drugs can be more evenly distributed.^[32]^ Patients who may benefit the most from HIPEC are those whose disease burden is limited to positive cytology and limited nodal involvement.^[33]^ So based on SC, we attempt to answer the question for the treatment strategies of GCPC clinically. It would be the first meta-analysis evaluating the efficacy and safety of HIPEC combined with SC for GCPC.

There are also some possible limitations to our review. There may be some heterogeneity across studies as the study designs are heterogeneous. To explore the possible sources of heterogeneity, we will perform a subgroup analysis. Therefore, the effects of various HIPECs on prognosis will also be analyzed. We aim to provide evidence-based suggestions for the clinical use of HIPEC.

## Author contributions

Song Zheng is the guarantor. Yidan Lu and Zheng Jin drafted the manuscript protocol. Yidan Lu designed the search strategy and provided statistical expertise. Zheng Jin, Yurong Bai, and Yangchen Sun contributed to the development of the selection criteria, article screening strategy, risk of bias assessment strategy, and data extraction criteria. All authors read, provided feedback and approved the final protocol.
